# A Novel Oxidative Stress Mediator in Acute Appendicitis: Thiol/Disulphide Homeostasis

**DOI:** 10.1155/2016/6761050

**Published:** 2016-08-23

**Authors:** Sefa Ozyazici, Faruk Karateke, Umit Turan, Adnan Kuvvetli, Huseyin Kilavuz, Burak Karakaya, Pınar Ozaltun, Murat Alısık, Ozcan Erel

**Affiliations:** ^1^Department of General Surgery, Adana Numune Training and Research Hospital, 01170 Adana, Turkey; ^2^1209 Sok., Akay Apt. No. 6, Palmiye Mahallesi, Mersin, Turkey; ^3^Department of Biochemistry, Faculty of Medicine, Yildirim Beyazit University, 01170 Ankara, Turkey

## Abstract

*Aim*. To investigate the role of a novel oxidative stress marker, thiol/disulphide homeostasis, in patients diagnosed with acute appendicitis (AA).* Methods*. In this study, seventy-one (43 male and 28 female) patients diagnosed with AA and 71 (30 male and 41 female) healthy volunteers were included. Age, gender, body mass index (BMI), haemoglobin (Hb), white blood cell (WBC), c-reactive protein (CRP), and thiol/disulphide homeostasis parameters (native thiol, total thiol, disulphide, disulphide/native thiol, native thiol/total thiol, and disulphide/total thiol ratios) were compared between the groups. Thiol/disulphide homeostasis was determined by a newly developed method by Erel and Neselioglu.* Results*. The native thiol, total thiol, and the native thiol/total thiol ratio levels were statistically significantly decreased in the AA compared with the control group (*p* < 0.001). Disulphide level and the ratios of disulphide/native thiol and disulphide/total thiol were higher in the AA group than in the control group (*p* < 0.001). There was a negative correlation of CRP with native thiol, total thiol, and native thiol/total thiol ratio while there was a positive correlation of CRP with disulphide/native thiol and disulphide/total thiol in the AA group. In the stepwise regression model, risk factors as disulphide/native thiol (OR = 1.368; *p* = 0.018) and CRP (OR = 1.635; *p* = 0.003) were determined as predictors of perforated appendicitis compared to the nonperforated group.* Conclusion*. This is the first study examining the thiol/disulphide homeostasis as a diagnostic aid in AA and establishing thiol/disulphide homeostatis balance shifted towards the disulphide formation due to thiol oxidation. Further studies are needed to optimize the use of this novel oxidative stress marker in AA.

## 1. Introduction

Acute appendicitis (AA) is the most common abdominal emergency worldwide that requires surgical intervention in the daily clinical practice of general surgery [1]. The diagnosis of acute appendicitis has been primarily made with clinical symptoms and physical examination. Since symptoms of AA overlap with other abdominal and gynecologic conditions, achieving an accurate diagnosis is still a clinical challenge for surgeons and there is a lack of adequate specific biomarkers for AA. To support the diagnosis, preoperative scoring systems such as the Alvarado score [2] were developed; however, these systems are rarely used. In clinical practice, the most commonly used laboratory tests for the diagnosis of AA are WBC and CRP. The nonspecific presentation and limited diagnostic accuracy of laboratory results often lead to an inadequate diagnosis, necessitating radiological imaging of the abdomen. Ultrasonography (US) and computed tomography (CT) scanning can enhance diagnostic accuracy in patients with suspected AA [3].

Although the pathophysiology of AA has been described in detail, the factors involving the progression of AA are still being investigated. Recently, it was found that one of these factors of interest is oxidative stress. The possible role of oxidative stress parameters in the progression of AA has been demonstrated in a few studies [4–6]. Some biochemical markers, such as those used to identify oxidative stress and inflammation, have been proposed as indicators of the presence and extent of acute appendicitis [5, 7]. It has been known that plasma thiols are physiological free radical scavengers and they may serve as an antioxidant function by several mechanisms. It has been known that the measurement of plasma total thiol and determining thiol/disulphide homeostasis are a good reflection of excess free radical generation in several diseases [8–10].

In this study, we hypothesized that oxidative stress plays a role in the pathogenesis of AA and we aimed to investigate a novel oxidative stress marker, thiol/disulphide homeostasis, in patients diagnosed with AA, as well as its correlation with other inflammatory markers such as CRP and WBC.

## 2. Materials and Methods

### 2.1. Study Population

This study was carried out in Adana Numune Training and Research Hospital General Surgery Clinic between April 2015 and December 2015. A total of 142 participants, consisting of 71 patients who underwent operations with diagnosis of AA over the age of 18 years and 71 healthy volunteers for the control group were included in the study. The control group consisted of healthy patients with similar demographic characteristics to the patient group and who have applied to our hospital with dyspeptic complaints but without a known chronic disease, smokers and alcohol users or drug users. Participants were also excluded from the study if they were pregnant or had a history of malignancy or abdominal trauma within 7 days of admission. Participants in the control group were included according to their reference sequence. The diagnosis of acute appendicitis was made with clinical symptoms and physical examination as well as laboratory tests and imaging techniques such as ultrasonography and computed tomography. This study has been designed in accordance with 2013 Brazil version of Helsinki Declaration and was approved by the local Ethics Committee. All participants have provided written consent. The principles of good clinical practices were followed during the study period.

### 2.2. Biochemical Parameters

Venous blood samples were taken to measure thiol/disulphide homeostasis parameters of all participants who were included in the study. In AA patients, blood samples were taken when they admitted to our clinic before surgical intervention. In healthy volunteers, blood samples were taken when they were admitted to our policlinic. After blood samples were quickly centrifuged at 1500 rpm for ten minutes, plasma and serum samples were separated. Serum samples have been stored at −80°C until all samples were obtained. Then the plasma samples tested for thiol/disulphide levels were shipped to biochemical laboratory of Ataturk Training and Research Hospital, Ankara, Turkey, after all samples were collected. Hemogram, biochemistry, and CRP levels of all participants were measured at the time they were enrolled in the study.

Thiol/disulphide levels were analyzed with a newly developed method by Erel and Neselioglu [11]. In summary, reducible disulphide bonds were first reduced to form free functional thiol groups. Unused reductant sodium borohydride was consumed and removed with formaldehyde, and all thiol groups including reduced and native ones were detected after reaction with 5,5′-dithiobis-(2-nitrobenzoic) acid. Half of the difference between total and native thiols provided the dynamic disulphide amount (-S-S). After the determination of native thiol (-SH) and disulphide (-S-S) amount, native thiol/disulphide ratio (-S-S-/-SH) was calculated. Laboratory staff performing the plasma thiol/disulphide homeostasis measurement analysis were blinded to the patients' clinical information and outcome; and results were not available to the treating physicians, study staff, or investigators during study period.

### 2.3. Statistical Analysis

Statistical Package for Social Sciences (SPSS) for Windows 20 (IBM SPSS Inc., Chicago, USA) program was used for statistical analyses. Kolmogorov-Smirnov test was used to determine the distribution of data. Continuous variables with normal distribution were given as mean ± standard deviation and continuous variables without normal distribution were given as median interquartile range [IQR]. Categorical variables were expressed as numbers and percentage. Continuous variables were compared with independent sample *t*-test, ANOVA, Mann Whitney *U* test, and Kruskal-Wallis *H* test where appropriate. Chi-square test and Fisher's exact chi-square test were used to compare categorical variables. The relationship between the numeric parameters was analyzed by Pearson and Spearman correlation analysis. Stepwise multivariable logistic regression analysis was used to identify independent predictors of AA risk. A *p* < 0.05 was considered significant for statistical analyses.

## 3. Results

A total of 142 individuals were evaluated in the study. No significant difference was observed between the AA and control groups with respect to age, Hb, and BMI (*p* > 0.05). The native thiol, total thiol, and the native thiol/total thiol ratio levels were statistically significantly decreased (*p* < 0.001) in the patients with AA compared with the control subjects. On the other hand, disulphide level and the ratios of disulphide/native thiol and disulphide/total thiol were higher in the AA group than in the control group (*p* < 0.001). The demographics and the thiol/disulfide homeostasis parameters of the patients and healthy controls are summarized in Table 1.

In the appendectomy group, patients were further divided into two categories: nonperforated appendicitis (*n* = 54) and perforated appendicitis (*n* = 17). There was no significant difference between the two groups in terms of sex, BMI, and Hb (*p* > 0.05). The levels of native thiol and total thiol (*p* = 0.003 and *p* = 0.005, resp.) as well as the native thiol/total thiol ratio (*p* = 0.04) were lower in the perforated group when compared to those of the nonperforated group. On the other hand, disulphide level and the ratios of disulphide/native thiol and disulphide/total thiol were higher in the perforated group than in the nonperforated group (*p* = 0.027, *p* = 0.001, and *p* = 0.001, resp.). The demographics and the thiol/disulfide homeostasis parameters of the patients in AA group are summarized in Table 2.

Table 3 shows the correlation analysis between thiol/disulphide homeostasis parameters and other characteristic features with study population. We determined a negative correlation of CRP with native thiol, total thiol, and native thiol/total thiol ratio, while we determined a positive correlation of CRP with disulphide/native thiol and disulphide/total thiol in the AA group. There was a negative correlation of age with total thiol and native thiol/total thiol in the AA group. There was a negative correlation of age with native thiol and total thiol in the control group.

In the stepwise regression model formed with risk factors such as age, sex, BMI, WBC, Hb, CRP, native thiol, total thiol, disulphide, disulphide/native thiol, disulphide/total thiol, and native thiol/total thiol, risk factors as disulphide/native thiol (OR = 1.368; *p* = 0.018) and CRP (OR = 1.635; *p* = 0.003) were determined as predictors of perforated appendicitis compared to the nonperforated group. It was determined that 1-unit increase in disulphide/native thiol rate predicts 1.368 times the risk of having perforated appendicitis and 1-unit increase in CRP level predicts 1.635 times the risk of having perforated appendicitis. These data are summarized in Table 4.

## 4. Discussion

Acute appendicitis is still a clinical challenge for physicians, as there is no single test that reliably differentiates AA from other causes of acute abdomen. Developing specific diagnostic tests for AA would both prevent unnecessary surgery and help to define the advanced stages of the disease requiring urgent intervention, such as perforated appendicitis, which is associated with increased morbidity and mortality. Several plasma markers have been investigated in order to improve the diagnosis of AA [12]. In addition to inflammatory markers, oxidative stress markers have recently become an area of interest for investigators. In the literature, experimental and clinical studies addressing the association between AA and oxidative stress are limited [5, 13, 14]. It has been reported that the serum levels of oxidative stress markers change in patients with AA, determining that the imbalance between oxidant and antioxidant defense systems may play a role in the pathogenesis of AA, which could be either etiologic or a result of inflammation [9, 13, 15]. Therefore, these serum markers may be used to aid the diagnosis of AA and to determine the extent of the disease.

As is known, thiols are a class of organic compounds that contain a sulfhydryl group (-SH), which is composed of a hydrogen and a sulphur atom attached to a carbon atom [16]. Those disulphide bonds can be reduced back to thiol groups; therefore, thiol/disulphide homeostasis is maintained [17]. Thiols contribute the major portion of the total antioxidants present in the body and play an important role in defense against reactive oxygen species [18] and also play critical roles in programmed cell death, detoxification, antioxidant protection, and regulation of cellular enzymatic activity [11, 19]. Recently, it is known that an abnormal thiol/disulfide homeostasis state is involved in the pathogenesis of various acute and chronic diseases [10, 11]. Measuring thiols in serum provides an indirect reflection of the antioxidative defense [10, 11, 18–20]. The measurement of dynamic thiol/disulphide first started by a new automated method developed by Erel and Neselioglu [11]. This study aimed to determine the status of dynamic thiol/disulphide homeostasis by this new method in AA. Furthermore, we investigated the correlation between thiol/disulphide homeostasis parameters and other clinical features in AA patients.

Previously, Yilmaz et al. [9] and Dumlu et al. [13] found decrease in thiol levels in appendicitis patients compared with controls. Under oxidative stress, disulphide level is expected to increase as thiol level decreases. However, this topic was not studied before in patients with AA. To the best of our knowledge, this is the first study that investigated thiol/disulphide homeostasis as a novel marker of oxidative stress in patients with AA and compared the results with healthy controls. In our study, we demonstrated that the levels of native thiol and total thiol and the native thiol/total thiol ratio are lower in patients with AA as compared to healthy individuals. Besides, we also demonstrated for the first time that disulphide level and disulphide/native thiol and disulphide/total thiol ratios, all of which are formed as a result of thiol oxidation, are higher in patients with AA than in the healthy controls. In other words, thiol/disulphide homeostasis was found to shift towards disulphide formation. Thiol/disulphide homeostasis parameters were similar as for the subgroup analysis of AA patients. We assume that this situation shows the higher level of oxidative stress in perforated appendicitis patients compared to that of nonperforated appendicitis.

In clinical practice, CRP and WBC values are used to predict the severity of inflammation in AA. Previous studies have shown that the greater the degree of appendicular inflammation, the greater the CRP value, reaching maximum values in cases of perforation [5, 7]. Similarly, our study has disclosed a significant increase in CRP in appendicitis progressed to perforation. Furthermore, correlation analysis showed a negative correlation of CRP with native thiol, total thiol, and native thiol/total thiol ratio, while a positive correlation of CRP with disulphide/native thiol and disulphide/total thiol was noted. In the stepwise logistic regression analysis formed with risk factors, it was determined that disulphide/native thiol and CRP are independent predictors of perforated appendicitis. Therefore, with the results of this study, we can say that when the increase of disulphide/native thiol ratio due to severity of inflammation and its positive correlation are considered together, it is possible to conclude that disulphide/native thiol ratio could be related with progression and used as a marker of disease activity. Although plasma thiol/disulphide homeostasis parameters cannot be used in the diagnosis of AA, a shifting towards disulphide in its value by time might be considered as a predictor of the progression of inflammation to the perforation in AA cases.

There are several limitations in this study that should be taken into consideration. First, this was a pilot study representing an initial investigation into the relationship between AA and thiol/disulphide homeostasis parameters. Second is inclusion of relatively small number of patients who were admitted to a single center. Other diagnostic aids such as procalcitonin and imaging techniques such as ultrasonography and/or computed tomography and Alvarado score have not been correlated with thiol/disulphide homeostasis parameters. Lastly, the plasma samples tested for the thiol/disulphide homeostasis were frozen and shipped to a single laboratory remote from our hospital rather than on-site using fresh plasma samples as would be done in clinical practice. Further longitudinal studies on a larger patient population are needed to determine whether alterations in thiol/disulphide homeostasis could be predictive risk factors for AA.

## 5. Conclusion

This study demonstrated that dynamic thiol/disulphide homeostasis shifted towards disulphide formation as a result of thiol oxidation in patients with AA. Prospective and randomized controlled trials are necessary to confirm the pathophysiologic role of thiol/disulphide homeostasis in AA. Further studies are required to optimize the use of this novel oxidative stress marker in conjunction with other established approaches.

## Figures and Tables

**Table 1:** The demographics and laboratory findings of AA and control groups.

Variables	Acute appendicitis	Control	*p* value
	(*n* = 71)	(*n* = 71)	
Gender, *n* (%)			
Male	43 (60.6)	30 (42.3)	0.04
Female	28 (39.4)	41 (57.7)	
Age (years)	32.4 ± 9.1	30.1 ± 8.9	NS
BMI (kg/m^2^)	21.7 ± 2.9	23.8 ± 3.1	NS
WBC (*μ*L)	13.5 ± 2.7	7.1 ± 1.5	<0.001
CRP (mg/L)	1.3 (18)	0.6 (3)	<0.001
Hemoglobin (g/dL)	13.6 ± 0.9	13.6 ± 1.1	NS
Native thiol (*μ*mol/L)	270.7 ± 68.1	386.5 ± 58.5	<0.001
Total thiol (*μ*mol/L)	300.6 ± 66.8	406.9 ± 60.2	<0.001
Disulphide (*μ*mol/L)	18.6 ± 4.5	10.5 ± 3.3	<0.001
Disulphide/native thiol (%)	7.3 ± 3.1	2.7 ± 0.9	<0.001
Disulphide/total thiol (%)	6.1 ± 2.2	2.5 ± 0.8	<0.001
Native thiol/total thiol (%)	87.2 ± 5.1	94.9 ± 1.6	<0.001

*p* < 0.05 statistical significance.

Parameters were expressed as mean ± SD and median (interquartile range (IQR)).

BMI: body mass index (kg/m^2^); WBC: white blood cell; CRP: C-reactive protein.

**Table 2:** The demographics and laboratory findings of patients in AA group.

Variables	Perforated appendicitis	Nonperforated appendicitis	*p* value
	(*n* = 17)	(*n* = 54)	
Gender, *n* (%)			
Male	13 (76.5)	30 (55.6)	NS
Female	4 (23.5)	24 (44.4)	
Age (years)	36.5 ± 8.5	31.1 ± 8.9	0.031
BMI (kg/m^2^)	22.5 ± 3.4	21.4 ± 2.7	NS
WBC (*μ*L)	14.6 ± 3.3	13.1 ± 2.4	0.044
CRP (mg/L)	7.8 (17)	1.2 (10.6)	<0.001
Hemoglobin (g/dL)	13.9 ± 1.1	13.6 ± 0.9	NS
Native thiol (*μ*mol/L)	228.3 ± 60.2	284.1 ± 65.3	0.003
Total thiol (*μ*mol/L)	261.2 ± 68.3	312.7 ± 61.9	0.005
Disulphide (*μ*mol/L)	21.5 ± 6.4	17.7 ± 3.3	0.027
Disulphide/native thiol (%)	9.6 ± 3.2	6.6 ± 2.6	<0.001
Disulphide/total thiol (%)	7.9 ± 2.2	5.6 ± 1.9	<0.001
Native thiol/total thiol (%)	84.1 ± 4.4	88.1 ± 4.9	0.004

*p* < 0.05 statistical significance.

Parameters were expressed as mean ± SD and median (interquartile range (IQR)).

BMI: body mass index (kg/m^2^); WBC: white blood cell; CRP: C-reactive protein.

**Table 3:** Correlation analysis between characteristics and laboratory findings with study population.

Variables	Native thiol		Disulphide		Total thiol		Disulphide/native thiol		Disulphide/total thiol		Native thiol/total thiol	
	*r*	*p*	*r*	*p*	*r*	*p*	*r*	*p*	*r*	*p*	*r*	*p*
*Groups*												
Control												
Age (years)	−0.384	0.001^*∗*^	−0.077	0.522	−0.378	0.001^*∗*^	0.125	0.297	0.131	0.277	−0.125	0.299
BMI (kg/m^2^)	−0.020	0.867	−0.106	0.378	−0.039	0.746	−0.133	0.268	−0.134	0.264	0.138	0.250
WBC (*μ*L)	0.166	0.166	0.194	0.104	0.174	0.147	0.033	0.785	0.038	0.754	−0.019	0.876
CRP (mg/L)	0.085	0.479	0.080	0.505	0.084	0.484	−0.016	0.893	0.003	0.979	0.035	0.774
Hemoglobin (g/dL)	0.169	0.159	−0.057	0.639	0.164	0.171	−0.078	0.520	−0.076	0.529	0.079	0.512

AA												
Age (years)	−0.161	0.181	0.101	0.401	−0.268	0.024^*∗*^	0.192	0.109	0.179	0.135	−0.292	0.013^*∗*^
BMI (kg/m^2^)	−0.224	0.061	0.119	0.323	−0.154	0.201	0.128	0.286	0.136	0.260	−0.230	0.053
WBC (*μ*L)	−0.212	0.075	−0.017	0.888	−0.114	0.345	−0.022	0.854	−0.003	0.977	0.079	0.512
CRP (mg/L)	−0.322	0.006^*∗*^	0.114	0.344	−0.377	0.001^*∗*^	0.302	0.011^*∗*^	0.313	0.008^*∗*^	−0.234	0.050^*∗*^
Hemoglobin (g/dL)	0.094	0.434	0.139	0.247	0.120	0.320	−0.084	0.484	−0.089	0.461	0.041	0.733

^*∗*^ *p* < 0.05 statistical significance.

BMI: body mass index (kg/m^2^); WBC: white blood cell; CRP: C-reactive protein.

**Table 4:** Regression analysis and independent risk factors predicting perforated appendicitis.

Variables	OR	95% CI		*p* value^*∗*^
		Lower	Upper	
Disulphide/native thiol	1.368	1.056	1.773	0.018
CRP	1.635	1.183	2.260	0.003

Nagelkerke *R* ^2^ = 0.586,  *p* < 0.001.

^*∗*^Age, sex, BMI, and laboratory findings are included in the stepwise logistic regression model.

^*∗*^ *p* < 0.05 statistical significance.

CRP: C-reactive protein.
